# Triptans and troponin: a case report

**DOI:** 10.1186/1750-1172-4-15

**Published:** 2009-06-18

**Authors:** Claudia R Weder, Markus Schneemann

**Affiliations:** 1 Department of Internal Medicine, University Hospital of Zürich, Rämistrasse 100, CH-8091 Zürich, Switzerland

## Abstract

This case report describes for the first time acute coronary syndrome in a 67-year old patient after oral intake of naratriptan for migraine. So far in the literature, only sumatriptan, zolmitriptan and frovatriptan have been described to cause acute coronary syndromes.

A 67-year old Swiss woman with thoracic pain after intake of 2.5 mg naratriptan presented with T-wave inversions in the ECG and a positive troponin-T at our hospital. Coronary angiography showed normal coronary arteries. Naratriptan-induced coronary vasospasms were thought to have caused the acute coronary syndrome.

Triptans should not be prescribed in patients with pre-existing coronary heart disease. However, triptans can also cause acute coronary syndromes in patients without coronary heart disease – as described in our case report. Severe or persistent thoracic pain after intake of triptans should therefore be investigated accordingly.

## Background

Triptans are an established treatment of acute migraine attacks[1]. By activating 5HT1B/1D-receptors they lead to vasoconstriction of cerebral blood vessels which are dilated during migraine attacks[2]. Moreover, they reduce secretion of vasoactive peptides and conduction of pain stimuli over the cerebral cortex[2]. Although triptans exert their therapeutic effect through cerebral vasoconstriction, studies have also documented vasoconstriction in the coronary, pulmonary, and systemic vasculature[3]. Under treatment with triptans in 1–7% thoracic pain occurs, which is mostly transient, mild and without lasting ischemia[4]. The incidence of myocardial infarction under triptans is estimated to be very low[4]. Most published case reports refer to myocardial infarction after the use of sumatriptan (subcutaneous [5-8,14] oral [9-12] and nasal[13] application), whereas both stenotic [6-8,12] and normal [9-11,13,14] coronary arteries could be found angiographically. Besides sumatriptan, acute coronary syndrome has been described after the use of zolmitriptan [15,16] and frovatriptan[17].

Here we report for the first time acute coronary syndrome in a 67-year old Swiss patient after oral intake of naratriptan for migraine. Migraine attacks stopped completely in our patient after implementation of the calcium blocker amlodipine as antihypertensive treatment. Different calcium blockers can be used for migraine prophylaxis (verapamil, diltiazem, nifedipine)[18], but are not recommended as first choice treatment. The efficacy of amlodipine as preventive migraine treatment has been reported in only 2 case reports so far [18,19].

## Case presentation

A 67-year old patient was submitted to our emergency unit because of thoracic pain. Her medical history was positive for migraine headaches for the last 45 years. The migraine was controlled satisfactorily with naratriptan, which the patient has been taking for the last five years on an as-needed basis. So far, the patient has never experienced any adverse event after taking naratriptan. The patient had a positive history for arterial hypertension and colon irritabile and had a regular medication of lisinopril 5 mg once daily, paracetamol 1 g once daily, magnesium 5 mg two times daily and cholestyramin 4 g once daily.

On the evening before hospital admission the patient suffered from an acute migraine attack with headaches and nausea so that she took 2.5 mg naratriptan orally as usual. Thereafter the headaches eased off. 30 to 60 minutes later the patient felt thoracic pain. During the following night the pain increased steadily with radiation to the neck. The next morning the patient went to see her family doctor. As the patient presented with T-wave inversions on the ECG in the first four precordial leads and a positive test for troponin the family doctor sent the patient to our hospital with the diagnosis of an acute coronary syndrome.

**Figure 1:**
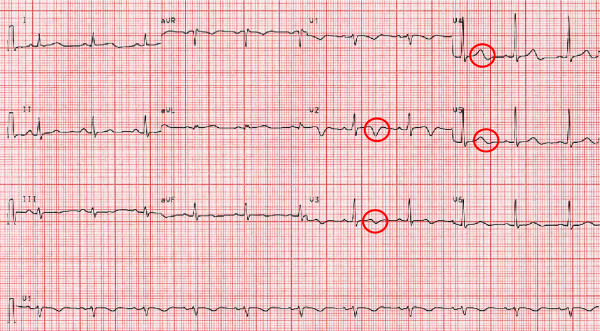
**Electrocardiogram with T-wave inversions in leads V1 to V4**.

**Figure 2:**
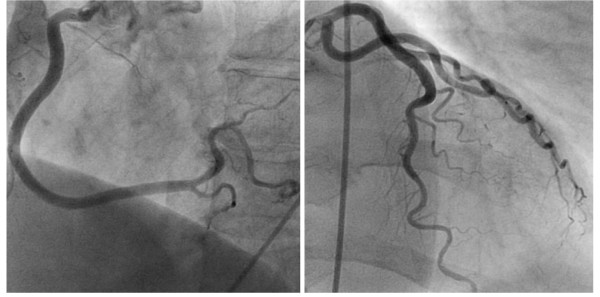
**Coronarangiography with patent coronary arteries**.

The ECG on admission showed T-wave inversions in leads V1 through V4 (figure 1). Creatine Kinase (CK) rose to a peak of 518 U/l (normal value < 167 U/l) and troponin-T to 0.42 ug/l (normal value < 0.1 ug/l). The patient was initially treated with acetylsalicylic acid 500 mg, clopidogrel 600 mg, heparine 5000 IU, oxygen and nitrates. Coronary angiography showed patent coronary arteries (figure 2) and ventriculography of the left ventricle a normal ejection fraction of 59%. Naratriptan was stopped as as-needed migraine medication and an antihypertensive treatment with the calcium blocker amlodipine was implemented. The patient was discharged in good condition after four days. Her cardiac status remained stable two years after the acute coronary syndrome. Moreover, the patient reported that she did no longer suffer from migraine attacks since taking amlodipine.

## Discussion

We postulated transient, prolonged coronary vasospasm caused by the vasoconstrictor properties of naratripan as the pathophysiological mechanism for the acute coronary syndrome in this patient without underlying coronary heart disease. The long duration of the patient's symptoms can be explained by the long half life of naratriptan, which is six hours. To prove our hypothesis of triptan-induced coronary vasospasm, a provocative testing with naratriptan could have been performed during the coronary angiography. However, as the history was very suggestive we abstained from this procedure. Besides the intake of naratriptan no other triggers for migraine were evident in this patient.

One important differential diagnosis in our patient is cardiac headache which is an extremely rare atypical presentation of coronary syndrome[20]. However, the presentation of our patient was more likely explained by triptan-induced coronary vasospasm as the patient was known for migraine for many years, migraine attacks had always a typical presentation, responded well to triptans and were not exertional. Moreover, the patient's actual chest pain occurred after taking naratriptan when the migraine had already eased off.

The fact that migraine attacks stopped completely after implementation of amlodipine as antihypertensive treatment can be explained by the vasodilatatory effects of amlodipine.

## Conclusion

Triptans should not be prescribed in patients older than 65 years of age and in patients with underlying coronary heart disease. But also patients without coronary heart disease may develop acute coronary syndromes after intake of triptans, as described in this case report. Severe or persistent thoracic pain after intake of triptans should be investigated promptly.

## Competing interests

The authors declare that they have no competing interests.

## Authors' contributions

CW was the major contributor in writing the manuscript. MS read and approved the final manuscript. Both CW and MS treated the patient.

## Consent

Written informed consent was obtained from the patient for publication of this case report and accompanying images. A copy of the consent form is available for review by the Editor-in-Chief of this journal.
